# Spatiotemporally Controlled Cardiac Conduction Block Using High-Frequency Electrical Stimulation

**DOI:** 10.1371/journal.pone.0036217

**Published:** 2012-04-30

**Authors:** Burak Dura, Gregory T. A. Kovacs, Laurent Giovangrandi

**Affiliations:** Department of Electrical Engineering, Stanford University, Stanford, California, United States of America; Georgia State University, United States of America

## Abstract

**Background:**

Methods for the electrical inhibition of cardiac excitation have long been sought to control excitability and conduction, but to date remain largely impractical. High-amplitude alternating current (AC) stimulation has been known to extend cardiac action potentials (APs), and has been recently exploited to terminate reentrant arrhythmias by producing reversible conduction blocks. Yet, low-amplitude currents at similar frequencies have been shown to entrain cardiac tissues by generation of repetitive APs, leading in some cases to ventricular fibrillation and hemodynamic collapse *in vivo*. Therefore, an inhibition method that does not lead to entrainment – irrespective of the stimulation amplitude (bound to fluctuate in an *in vivo* setting) – is highly desirable.

**Methodology/Principal Findings:**

We investigated the effects of broader amplitude and frequency ranges on the inhibitory effects of extracellular AC stimulation on HL-1 cardiomyocytes cultured on microelectrode arrays, using both sinusoidal and square waveforms. Our results indicate that, at sufficiently high frequencies, cardiac tissue exhibits a binary response to stimulus amplitude with either prolonged APs or no effect, thereby effectively avoiding the risks of entrainment by repetitive firing observed at lower frequencies. We further demonstrate the ability to precisely define reversible local conduction blocks in beating cultures without influencing the propagation activity in non-blocked areas. The conduction blocks were spatiotemporally controlled by electrode geometry and stimuli duration, respectively, and sustainable for long durations (300 s).

**Conclusion/Significance:**

Inhibition of cardiac excitation induced by high-frequency AC stimulation exhibits a binary response to amplitude above a threshold frequency, enabling the generation of reversible conduction blocks without the risks of entrainment. This inhibition method could yield novel approaches for arrhythmia modeling *in vitro*, as well as safer and more efficacious tools for *in vivo* cardiac mapping and radio-frequency ablation guidance applications.

## Introduction

Electrical excitation of cardiac cells and the mechanisms involved have been studied in great detail in the past century [1]. The technique has become a standard for cardiac electrophysiology and the basis for many therapies (e.g. [2]). However, electrical *inhibition* of cardiac activity – the ability to prevent cells from further generating an action potential (AP) – has not been investigated to the same extent [3]–[9] and has not yet found many practical applications. Inhibition of heart cells in animal models [3] and humans [4], [5] has been demonstrated using isolated [4] and trains [3] of subthreshold stimuli applied during the refractory period. Subthreshold stimuli have been shown to lengthen refractoriness [4], rendering the local tissue unresponsive to subsequent beats. Although promising results have been reported *in vivo* on the treatment of reentrant arrhythmia [2], inhibition efficiency of subthreshold protocols heavily depends on the timing (stimulation delivered during refractory period) and amplitude of the stimuli (narrow range of effective amplitudes). In particular, the amplitude condition imposes operational constraints, including limited extent of inhibition within the tissue [3]. Thus far, stimulus trains with amplitudes beyond the subthreshold range have mostly been shown *in vivo* to produce capture [6], [10], sometimes leading to fibrillation [10].

In some cases, inhibition by extension of action potential duration (APD) has also been achieved using low-frequency suprathreshold alternating current (AC) stimulation (20–60 Hz), as revealed in computer simulations [7], [8] and limited experimental work, both *in vivo* [9], [11] and *in vitro* [9], [12]. However, these studies also showed entrainment of the tissue, and revealed a multimodal response to increasing stimulus amplitude – no effect, single AP, multiple APs and single AP with prolonged plateau phase (i.e., extension of APD). Other work employing AC fields with similar frequency ranges also yielded induction of ventricular fibrillation (VF) [10], [13], continuous entrainment [14]–[16] and hemodynamic collapse [14]–[16] without observing any extended APD. In a concurrent study recently published, Tandri, *et al.* have shown the ability of sinusoidal electric fields in the 20 Hz–2 kHz frequency range to produce reversible conduction blocks in several models (including monolayers and whole hearts) through inhibition of excitability by prolonged APD [9]. Using field stimulation of the whole culture or organ, complete conduction blocks were achieved for durations of up to one second. The authors further demonstrated the effectiveness of these blocks in terminating reentrant arrhythmia. However, they did not observe inhibition above 2 kHz for the field amplitudes tested. Under 2 kHz, they consistently found entrainment for field amplitudes below blocking thresholds.

This sensitivity to amplitude has critical implications for applications in which the field (or current) might not be uniform. In such cases, while inhibition might be reached in regions of highest amplitudes, regions subjected to lower amplitudes may be entrained, potentially contributing to harmful side effects (e.g., fibrillation). Such non-uniformities would be expected in most clinical cases where the electrodes are much smaller than the human heart, such as with catheter tips or pacemaker and implantable cardioverter-defibrillator leads for instance.

In a single-cell modeling study of AC stimulation up to 50 Hz, Meunier *et al.* have shown that the multimodal tissue response to AC stimulation switches from four amplitude-dependent modes to three modes above 10 Hz (no response, multiple APs, single prolonged AP) [7]. Most significantly, the authors showed that the amplitude window for generation of multiple APs decreases with increasing frequencies as well. Based on the results of Meunier *et al.*, we hypothesized that at sufficiently higher frequencies, the amplitude window for multiple APs would disappear, and that the tissue response would become binary – no response or single prolonged APs – thereby avoiding entrainment by multiple APs. Here, we show in cultures of HL-1 cardiomyocytes that there exists indeed a frequency threshold above which the cells exhibit this binary response to stimulation amplitude, devoid of the entrainment observed at lower frequencies.

## Results

### Characterization of conduction block

**Figure 1:**
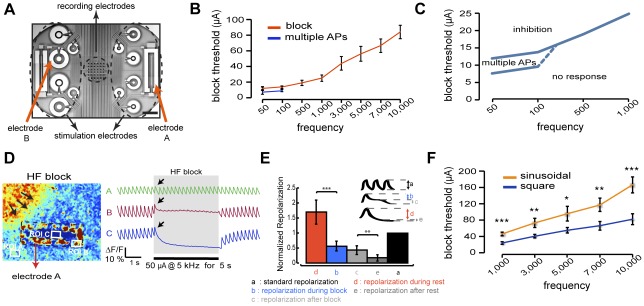
Characterization of suprathreshold AC inhibition and conduction block. A. Microelectrode array. Scale bar, 500 µm. B. Relationship between blocking threshold and frequency. C. Close-up (based on B) of the cell response to low frequency AC stimuli, highlighting the region of entrainment (multiple APs) before full response is reached. The dotted line is extrapolated. D. Left, representation of blocking electrode A with regions of interest (ROIs) for Ca^2+^ imaging. Right, example of Ca^2+^ recordings from ROIs during blocking experiments. Arrows point to the action potential generated at onset of inhibition stimulus. Black bar represents the inhibition duration. E. Repolarization analysis indicating prolonged action potentials for inhibited cells. Repolarizations are normalized with respect to standard repolarization during regular action potentials. (Error bars, s.d.; *n* = 9 cultures; *** indicates *p*<0.001; ** indicates *p*<0.01). F. Comparison of inhibition thresholds (peak-to-peak) between sinusoidal and square waveforms. (B, C and F: error bars, s.d.; *n* = 6 cultures; µA, microamperes; * indicates *p*<0.05; ** indicates *p*<0.01; *** indicates *p*<0.001.).

We first evaluated the response of HL-1 monolayers cultured on microelectrode arrays (Fig. 1A) to AC electrical stimulation between 50 Hz and 10 kHz using Ca^2+^ imaging and extracellular recordings. At low stimulation frequencies (50–100 Hz), we observed a multimodal response from the tissue (Fig. 1B,C). At very low amplitudes, AC stimuli did not elicit any response from the tissue. As the amplitudes rose, AC stimuli first evoked multiple irregular APs (4 out of 6 cultures, >20 trials), often resulting in the disruption of the normal pacing activity or a change in the origin of the wavefront (pacemaker site). Upon cessation of the stimuli, some cultures showed transient ectopic beating before following the pacing stimuli. Finally, above a certain threshold, a single AP was generated (onset response), after which the electrical activity was suppressed without interfering with the spontaneous activity in the non-stimulated areas. These results are consistent with reports by Antoni *et al.* [12] and recently by Tandri *et al.* [9], and the irregular multiple APs generated before reaching suppression thresholds could explain the induction of VF, multiple APs and hemodynamic collapse without inhibition observed in prior studies [13]–[16].

In contrast, AC stimulation at higher frequencies (≥500 Hz) resulted in a binary response. At low amplitudes, AC stimuli did not cause any response. At higher amplitudes, AC stimulation never induced multiple APs, but always generated suppression of the electrical activity after an onset response (6 out of 6 cultures; >50 trials), without influencing the propagation in non-stimulated areas (Fig. 1B,C). During the application of inhibition stimuli, cells overlying the electrodes showed prolonged depolarizations and elevated intracellular Ca^2+^ levels compared to typical AP Ca^2+^ transients on regions outside the electrode (Fig. 1D,E). The prolonged depolarizations prevented the subsequent stimuli from depolarizing the tissue, hence producing local inhibition and conduction block.

The single AP generated at the onset of the AC stimulation never caused a shift of the pacemaker sites or more than one skipped beat in paced cultures (due to the asynchronous onset of the inhibition stimulus). The reliability and controllability of the block were tested using varying electrode sizes (from 0.06 to 0.4 mm^2^) and stimuli durations (from 5 s to 300 s). The block was systemically induced and maintained in the cells overlying the electrodes for the duration of the stimulus (*n*>50 cultures; >200 trials). The block had rapid onset (within a beat) and rapid reversibility (within a beat), and was sustainable for long durations (tested up to 5 minutes; *n* = 7 cultures). Further characterization showed an increase in inhibition threshold with increasing AC stimulation frequency (Fig. 1B,C; see Fig. S1 for corresponding charge densities). Square waveforms required smaller amplitudes on average for suppression of activity compared to sinusoidal waveforms (Fig. 1F). Although statistical significance was consistently observed for peak-to-peak stimulus amplitudes, root-mean-square (RMS) amplitudes did not reach statistical significance for all frequencies.

**Figure 2:**
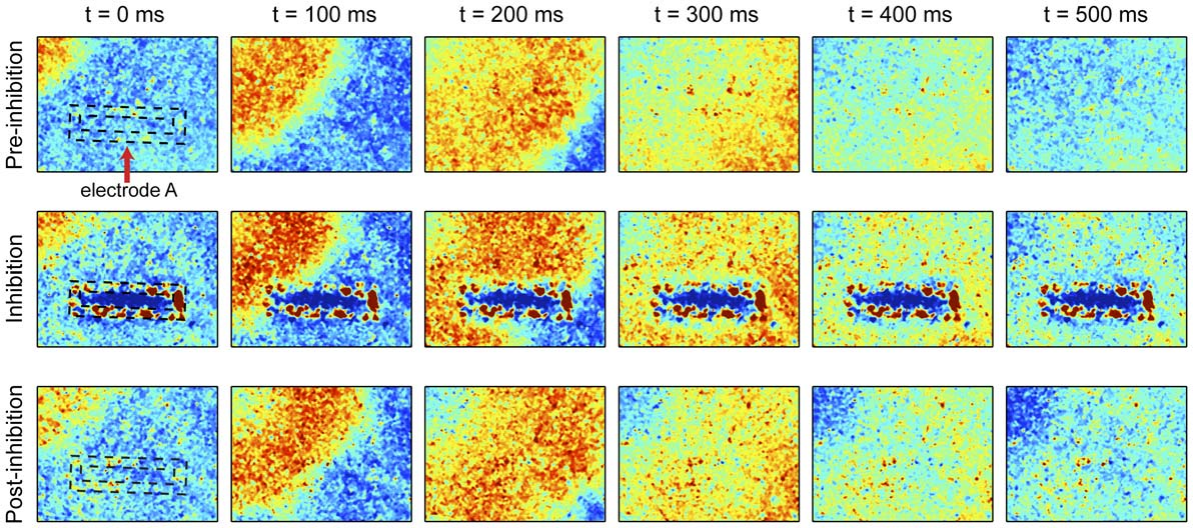
Demonstration of spatiotemporally controlled electrical conduction block (50 µA_p-p_, 5 kHz). Time-lapse of Ca^2+^ fluorescence showing propagation of electrical activity before, during and after application of the inhibitory stimulus. White dotted lines highlight the location and geometry of blocking electrode (electrode A in Fig. 1A,D). Red indicates high Ca^2+^ concentration, blue low.

The spatiotemporal patterning of local conduction blocks was best demonstrated with a large electrode enclosing a rectangular area, momentarily blocking the depolarization wave and preventing it from reaching and depolarizing the inner region (Fig. 2; Movie S1). Intracellular Ca^2+^ levels in this protected region were significantly reduced during the blocking, reflecting the quiescent state of these regions (Fig. 1D,E). Overall, the combination of suprathreshold amplitudes and high frequencies thus leads to an effective inhibition and a well-defined response of the cells. In particular, high frequencies are critical to avoiding potential entrainment observed at lower frequencies and associated with amplitude gradients surrounding stimulation electrodes (Fig. 1C).

### Control of conduction path

**Figure 3:**
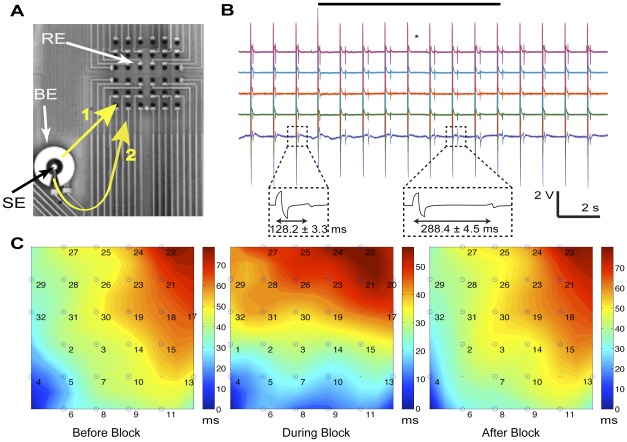
Guidance of conduction path using high-frequency suprathreshold AC stimuli. A. Microelectrode array used in the experiment. Yellow arrows labeled 1 and 2 represent the conduction paths before and during the block, as derived from the isochrone maps in (C). SE, stimulation electrode used for pacing cells; BE, blocking electrode; RE, recording electrodes. B. Electrical recordings showing the change in conduction path during blocking by the increased time delay between stimulation pulse and LATs. Black bar represents the block duration. (* denotes one missed beat during the experiment). C. Isochrone maps revealing the direction change in conduction path.

In addition to the spatiotemporal control of inhibition, we also show its application in controlling conduction path as an illustration of the potential of this technique for *in vitro* assays. The cardiomyocytes were paced using one of the stimulation electrodes at 60 beats per minute (bpm) and blocking was performed with the surrounding incomplete annular electrode (Fig. 3A). Blocking AC stimulation was performed at 5 kHz at the inhibition threshold amplitude. Without the blocking stimulus, evoked electrical activity spread radially from the pacing electrode to other regions. From electrical recordings, the initial propagation path was estimated using the local activation times (LATs) of the recorded APs (path labeled 1, Fig. 3A,C). Upon turning on the blocking AC stimulus, the recordings showed increase in the average time delay (from 128.2±3.3 ms to 288.4±4.5 ms, averaged over four beats) between the pacing pulse and average LATs on the recording electrodes (Fig. 3B). As determined by the isochrone maps, the initial propagation path was blocked and depolarization originating from the pacing electrode was propagated to other regions through the unblocked opening of the incomplete annular electrode (path labeled 2, Fig. 3A,C). Termination of the blocking signal restored the propagation of activity in the initial path (Fig. 3A,C). This ability to pattern conduction pathways via the blocking mechanism presented could help generate *in vitro* models reproducibly replicating complex conduction patterns such as spiral and reentrant waveforms for the study of fibrillation.

### Single-cell simulations

**Figure 4:**
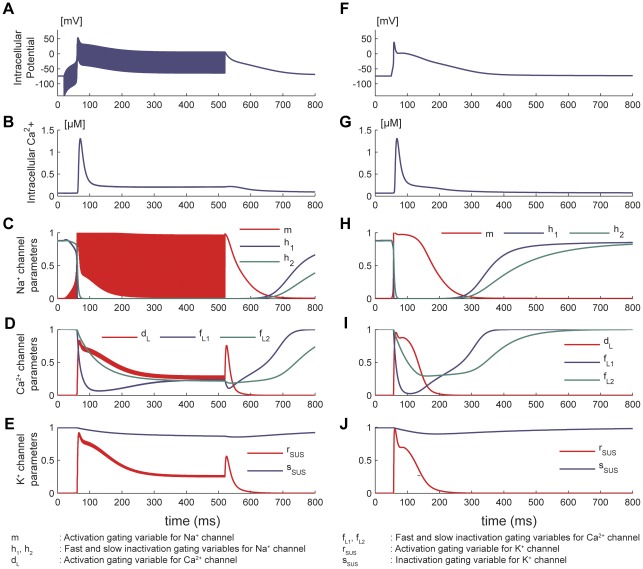
Single-cell simulation results of suprathreshold AC inhibition compared to normal action potential. A–E. Prolonged action potential. Simulated responses to 1 kHz square wave applied between *t* = 10 ms and *t* = 510 ms. Prolonged action potential revealed by membrane potential (A, F) and intracellular Ca^2+^ levels (B, G). C. Inactivation of Na^+^ channels (during the block, fast and slow inactivation gating parameters – *h*_1_ and *h*_2_ – decreased to zero; activation gating variable *m* oscillated between 0 and 1 in comparison to resting values in H). D. Prolonged activation of inward Ca^2+^ channel (during the block, fast and slow inactivation gating parameters – *f*_L1_ and *f*_L2_ – remained lowered while activation gating variable *d*_L_ remained high compared to resting values in I). E. Prolonged activation of outward K^+^ channel (during the block, inactivation gating variable *s*_SUS_ lowered to 0.9 while activation gating variable *r*_SUS_ plateaued around 0.3 in comparison with resting values in J). F–J. Normal action potential. Simulated responses to a stimulus of 10 ms duration applied at *t* = 40 ms.

To investigate the possible ionic mechanisms behind the high-frequency block, we performed computer simulations based on a single-cell model [17]. Results of simulations at 1 kHz revealed extended depolarizations and elevated Ca^2+^ levels (Fig. 4A,B) similar to experimental results. At the membrane level, these simulations showed the inactivation of Na^+^ channels (Fig. 4C) and prolonged activation of sustained K^+^ (*I*_sus_) and L-type Ca^2+^ (*I*_Ca,L_) channels (Fig. 4D,E, Fig. S5) for the duration of the stimulus with respect to their values during typical APs (Fig. 4F–J). The prolonged depolarization might thus be explained by the counteraction of the outward K^+^ repolarization current by the inward Ca^2+^ current – sustained for the duration of the stimulus – while the suppression of further depolarization might be related to the inactivated Na^+^ channels. Further experimental data will be needed to test these hypotheses. Thresholds for sinusoidal waveforms were higher compared to square waveforms in simulations, matching experimental observations (Table S1, S2).

Simulations also led to a similar multimodal response with increasing stimulus amplitudes as observed experimentally (Fig. S2, S3, S4, S5). For frequencies below 25 Hz, the response of the cell switched from no response to single AP, and finally to multiple APs with increasing amplitudes. Prolongation of APD was not observed (Table S1). This is also in good agreement with the experimental results from Tandri *et al.* [9], in which the authors did not observe prolonged APs for frequencies below 20 Hz. For frequencies above 25 Hz, single-cell simulations also revealed prolonged AP response at higher stimulus amplitudes. However, simulations did not reproduce the binary response at high frequencies observed in experiments, and always resulted in an amplitude-dependent multimodal response (Table S1).

## Discussion

### Implications of present findings

We have demonstrated a reliable, responsive and reversible cardiac conduction block, which is spatially and temporally controlled by electrode geometry and stimulus duration respectively, and is immune to potential entrainment observed at lower frequencies. Compared to subthreshold stimulation, this method offers a larger operational range and enables the inhibition of a greater volume of cardiac tissue. Indeed, subthreshold stimulation is bound in amplitude by a lower threshold below which inhibition does not occur, and a higher threshold set by the risk of entrainment. This results in a narrow range of operation, limited spatial extent, and risks of uneven inhibition or entrainment due to small variation of thresholds across the tissue. The AC stimulation presented here, on the other hand, relies on larger, suprathreshold amplitude stimuli, extending the spatial reach, and increasing robustness against such local threshold variations.

We have also shown that the use of high-frequency stimuli eliminates the risk of entrainment observed at lower frequencies and associated *in vivo* with fibrillation and hemodynamic collapse. For the atrial cells used in this study (HL-1), multiple APs were observed for frequencies below 100 Hz, but not for frequencies greater than 500 Hz. This suggests that a critical threshold frequency for avoiding the multiple-AP response lies between 100 and 500 Hz. These results are supported by the computer model studies of Meunier *et al.* which show a monotonic decrease of the amplitude window leading to the generation of multiple APs with increasing frequencies [7]. Here, we demonstrated that at sufficiently high frequencies, this window disappears completely, avoiding the multiple AP response altogether.

In the recently published study by Tandri *et al.* on cardiac inhibition using AC field stimulation [9], the authors described a tissue response of rat neonatal ventricular myocyte monolayers that is consistent with our results. They found an inhibition threshold increasing with frequency, although they did not observe inhibition at frequencies above 2 kHz for the amplitudes tested (up to 11 V/cm). It is very likely that the inhibition thresholds at these frequencies were simply higher, following the frequency-dependence observed at lower frequencies. They also observed occurrences of multiple APs (called “field evoked activity”) at sub-threshold amplitudes, as we have seen for low frequencies. However, this field evoked activity was reported for frequencies all the way up to 2 kHz. The threshold frequency for a binary response might have been above 2 kHz, but the authors did not test higher stimulation amplitudes to observe inhibition beyond this frequency. While this threshold frequency would be higher than the one observed in our study, it could be explained by the difference in tissue types (rat neonatal ventricular vs. mouse-derived atrial myocytes) and their respective electrophysiological properties.

To date, inhibition by prolonged APD has been demonstrated for durations up to a few seconds. Using HF stimulation, we have been able to generate reversible inhibition for up to 5 minutes. While short durations (seconds) have already been proven effective in termination of arrhythmia or ablation guidance [2], [9]–[11], [18], [19], the ability to block conduction for longer durations provides further flexibility in deployment. This might prove especially useful in cardiac mapping, RF ablation guidance or *in vitro* models, where longer durations might be desirable (increased time for assessing impact of inhibition) or even necessary (sustained conduction blocks for reentry models). Altogether, these various properties make this method of inhibition readily applicable *in vitro*, and could lead to a safer, more practical approach for *in vivo* and clinical applications.

High-frequency stimulation provides further advantages for integration in electrophysiology systems. By using stimuli of higher frequencies than the measured potentials (typical upper limit of 1–2 kHz), the artifacts caused by this type of stimulation can be filtered out, thus allowing simultaneous recording of the tissue activity during stimulation [20]. This allows for real-time confirmation of the effectiveness of the inhibition, for instance, or for application in cardiac mapping, effectively alleviating the need for further optical measurement methods (Ca^2+^ or voltage-sensitive fluorescent dyes). In addition, the lower impedance of metal electrodes at high frequencies means that for a similar stimulation current, the electrode voltage will be lower for a higher frequency stimulus, thereby reducing irreversible chemical reactions and tissue damage [20], [21].

In recent years, the introduction of light-activated ion channels and their expression in neurons has given rise to a new field – optogenetics – enabling precise control of electrical activity using light [22], [23]. The functionality of optogenetic tools has also been explored in cardiac cells, and inhibition of cardiac activity by optical means has been recently demonstrated [24]. However, such methods require expression of exogenous channel proteins by gene therapy, rendering this method currently impractical for clinical use. In contrast, the proposed electrical inhibition protocol does not present such challenges, and is in fact readily compatible with existing stimulation hardware and delivery methods (microelectrodes, catheters, leads).

### Mechanisms of inhibition

Single-cell computer simulations suggest that continuous high-frequency AC stimulation leads to sustained elevated membrane potential, resulting in prolonged sodium channel inactivation and inhibition. While the optical Ca^2+^ measurements during inhibition support our simulation results of elevated intracellular Ca^2+^ and suggest elevated membrane potentials, they did not allow an absolute determination of these potentials. However, Tandri *et al.*, using a similar stimulation protocol and calibrated voltage-sensitive dyes, estimated the resulting elevated potential between −48 and 15 mV, which is typically above Na^+^ channel activation thresholds [9]. Our simulation results are thus also in good agreement with the experimental data presented by Tandri *et al.*

It is noteworthy that we were able to reproduce most of the experimental observations (depolarization, prolonged AP, frequency and amplitude dependences) with a single-cell model, strongly supporting membrane and channel mechanisms as the primary components of HF inhibition. An exception is the binary response at high frequencies, observed in experiments, but not reproduced in simulations. It is likely that this frequency-dependent phenomenon ensue from channel characteristics not fully described by the model used (for instance, no time constant is associated to inward rectifier K^+^ channels). Cell-to-cell interactions, not taken into account by such model, may also play a role in this response.

An important aspect of this inhibition method pertains to the onset response – in effect a depolarization induced by high-frequency AC stimulation. While AC stimuli of low frequencies (<20 Hz) are able to depolarize the cell on their first phase, they generally fail to do so at higher frequencies due to the lower charge delivery per phase. At these higher frequencies, another study by the authors [20] suggests that stimulation of cells is based on an asymmetrical response of the membrane to AC signals (primarily from the Na^+^ and K^+^ channels), leading to an integration of sub-threshold phases until the depolarization threshold is reached (*Gildemeister effect*) [15], [16], [21], [25]–[27]. Such integration was previously demonstrated for neurons by Bromm *et al.* [26] and Shoen *et al.* [21], with an inward rectification by Na^+^ channels as the main underlying mechanism. The experimental study by Bromm *et al.* also pointed to a slight anomalous inward rectification by K^+^ channels [26].

In light of the simulation results provided in this study and the previously published results cited above, we can summarize the proposed inhibition mechanism as follows: the AC stimulation raises the membrane potential through the integration of sub-threshold pulses until it triggers an AP, termed onset response. As long as the AC stimulus is applied, the outward repolarization current due to K^+^ ions is then counter-balanced by the inward Ca^2+^ current, thereby keeping the membrane potential elevated. At such elevated potentials, the sodium channels remain inactivated, resulting in a prolonged refractory state (inhibition).

### Application in *in vitro* studies

The ability to generate arbitrary patterns of conduction by selective blocking in cardiac cell cultures is of direct interest for *in vitro* conduction and arrhythmia models. Traditional techniques usually require permanent, physical patterning of the cells, through surface modification [28] or physical constrains [29]. In contrast, this method induces reversible, reconfigurable blocks without any particular tissue preparation. An important application area would be the assessment of susceptibility to reentrant arrhythmias – a factor in risk assessment and stratification as well as a significant drug target. Such a reentry assay could use rings of various diameters and electrically defined in the target tissue (cell culture or tissue slice) to measure key parameters defining this susceptibility (e.g., conduction velocity, effective refractory period, cycle length, wavelength [30]), in a rapid and reproducible manner.

### Clinical implications

Clinical applications of inhibition have been demonstrated in previous studies using STS, notably for the termination of ventricular tachycardia [18] and atrioventricular node reentrant tachycardia [2]. However, the spatial and temporal limitations of STS strongly impacted the usability and performances of this approach [31], and have precluded its use for the management of arrhythmias.

Recently, a proof-of-concept for termination of reentrant arrhythmia by inhibition of whole hearts by HF AC stimulation has also been shown by Tandri et al. [9]. The experiments, using field stimulation of whole hearts, clearly demonstrated the potential of HF AC stimulation to effectively alter conduction patterns *in vivo*, but also raised questions about safety of deployment. Both Tandri's and our study showed that stimulation at sub-inhibition thresholds can induce entrainment for low frequencies, including all frequencies used in Tandri's study. As previously discussed, such sub-threshold amplitudes are likely to occur in clinical settings due to the relative size of electrodes (pads, leads) compared to the human heart and resulting amplitude gradients. In cases of localized inhibition (for RF ablation guidance or cardiac mapping, for instance), such gradients are simply inevitable. In any case, it would be highly desirable if the possibility of entrainment could be completely avoided. By relying on stimulation frequencies leading to a binary response (no effect/inhibition), the inhibition method and conduction blocks demonstrated in this work could therefore lead to potentially more efficacious and safer tools for *in vivo* use.

This method could also offer a more practical approach to radio-frequency (RF) ablation guidance – where application of STS has also been demonstrated [19] – owing to the inherent capability of suprathreshold stimuli to affect larger volumes of tissue. By enabling a deeper inhibition under the catheter, temporary blocks could be reliably tested before permanent ablation, as it is already possible with cryogenic ablation. Nevertheless, this inhibition method will require further experimental and clinical studies to establish its full potential.

### Study limitations

Our results and conclusions were based on experiments performed on mouse-derived atrial cell cultures. While directly usable in applications such as *in vitro* arrhythmia modeling, the translation of these results to *in vivo* and clinical applications will require further studies, notably with respect to the role of tissue structure (volume vs. single-cell layer), tissue type (ventricular vs. atrial), and species (human vs. mouse).

This work also only considered single-cell model simulations in the investigation of the inhibition mechanisms. These single-cell simulations managed to reproduce most of the experimental observations, and provided important insights on possible mechanisms for HF inhibition. However, they did not reproduce the binary response observed in experiments, which may point at a limitation of the model in its ability to reproduce some high-frequency characteristics of the channels. Single-cell models also do not capture the complexity of two-dimensional tissues, such as spatial gradients of applied potentials, virtual electrode effects and cell-cell coupling, all of which may affect inhibition thresholds to some extent. Adaptation of the model and extension to 3D modeling will likely be necessary in order to increase its predictive power.

### Summary

This work demonstrates the ability of high-frequency alternating currents to inhibit excitation in cardiomyocytes, produce reversible conduction blocks and guide propagation wavefronts *in vitro*. The data further reveal that above a critical frequency, cardiac tissue exhibits a highly desirable binary response to amplitude (no effect/inhibition), as opposed to an entrainment-prone, multimodal response at lower frequencies.

This electrically controlled inhibition method complements the well-known electrical excitation, providing together a powerful toolset for cardiac electrophysiology research, and could yield novel approaches for arrhythmia modeling *in vitro* or clinical applications such as radio-frequency ablation guidance.

## Materials and Methods

### Microelectrode array instrumentation

Microelectrode arrays (MEAs) used in this study consisted of 6×6 array of platinum electrodes with 22 µm diameters and a pitch of 100 µm (Fig. 1A), with additional larger electrodes on two sides for electrical stimulation [32]. A unipolar configuration was used for both recording and stimulation, with an oversized platinum electrode serving as common ground. Signals from the MEA were amplified by a custom amplifier system with a two-stage gain of 60 dB, a 4 Hz first-order high-pass cut-off, and an eighth-order low-pass cutoff at either 1.5 or 3 kHz, as previously reported [33]. 32 channels (four corner electrodes excluded) from the amplifier board were digitized with 12-bit resolution at 10 ksps and acquired by a custom designed visualization and extraction tool, written in MATLAB® (The MathWorks, Natick, MA, USA).

Isochrone maps were used to analyze conduction patterns. These maps were constructed based on the position and local activation time (LAT) of each electrode. LATs were defined as the point of maximum negative slope of an extracellular AP (minimum of the derivative).

### Cell culture protocols

The HL-1 mouse atrial cell line [34], generously provided by Dr. William Claycomb (Louisiana State University Health Science Center, New Orleans, LA, USA), was used in all experiments. The cells have a maximal diastolic potential of −68.8±1.6 mV with an overshoot of +15.3±1.9 mV and an AP waveform characteristics of atrial myocytes [35]. Extensive characterization has demonstrated how similar HL-1 cells are to primary cardiomyocytes [36].

Cells were cultured in T25 flasks at 37°C and 5% CO_2_ in culture medium consisting of Claycomb media (Sigma, St. Louis, MO, USA), supplemented with 10% fetal bovine serum (Invitrogen, Carlsbad, CA, USA), 100 µM norepinephrine (Sigma), 100 units/ml penicillin-streptomycin (Invitrogen), and 4 mM L-glutamine (Invitrogen) in a humidified chamber. After cells reached confluency and started beating, they were seeded onto MEAs as described previously [32]. Briefly, prior to seeding the cells, the MEAs were sterilized with 70% ethanol, rinsed with PBS (pH 7.2, Invitrogen), and coated with an adhesion-promoting solution containing 12.5 µg/ml fibronectin (Sigma) and 0.02% gelatin (VWR, Radnor, PA, USA) and stored in a 37°C incubator overnight. Cells were rinsed with PBS then trypsinized using 1.5 mL 0.05% trypsin/EDTA (Invitrogen) in incubator for 10 min. Trypsinization was stopped by adding 4.5 mL of medium followed by centrifugation (5 min, 1000 g). The supernatant was removed and the pellet was re-suspended in 5 mL media. The gelatin/fibronectin solution was aspirated from the arrays and replaced with 50–100 µL cell suspension. After one hour of settling time, chips were filled with 2 mL of medium. Medium was changed daily. Experiments were performed after cells reached confluency, usually 2–4 days after plating. The cultures were viable until 7–10 days following plating, depending upon plating density.

### Electrical stimulation protocols

The blocking effects of AC stimulation were determined in spontaneously beating cultures or paced cultures (if not spontaneously beating). In the latter case, one of the large stimulation electrodes on each side of the array (Fig. 1A) was used for pacing at 60 bpm. Overall, beating rates ranged between 60 and 120 bpm. For the investigation of stimulation parameters, current stimulation through an annular electrode (electrode B in Fig. 1A; area 0.2 mm^2^) was used in all experiments. The stimulations were carried out at eight distinct frequencies distributed between 50 Hz and 10 kHz using both square and sinusoidal waveforms. At each frequency, the peak-to-peak amplitude was increased in 1 to 5 µA steps beginning from 1 µA (corresponding current density 5 µA/mm^2^) until inhibition was observed. In experiments evaluating the reliability and controllability of conduction blocks (Fig. 2), 5 kHz square waveform blocking stimuli were applied to either an annular electrode (electrode B in Fig. 1A) or a large electrode enclosing a rectangular area (electrode A in Fig. 1A). In all experiments, current-controlled stimulation was performed using a custom-made voltage-controlled current source, driven by a function generator (DS360, Stanford Research Systems, Sunnyvale, CA, USA).

### Ca^2+^ imaging

Cells cultured on the MEAs were incubated with a 1∶1 mixture of the Ca^2+^-sensitive dye Fluo-4 Direct™ (Invitrogen) and Claycomb culture medium at 37°C for 30 min. The mixture was aspirated and replaced with 1 ml Claycomb culture medium before measurements. Calcium activity was recorded on an upright microscope (BX60M, Olympus, Center Valley, PA, USA) with a ×10 objective. Fluo-4 was excited using a mercury lamp through a 450–480 nm bandpass excitation filter. Fluorescence was collected through a high-pass emission filter at 515 nm. Frames were collected using a cooled CCD camera (Retiga-2000R, QImaging, Surrey, BC, Canada) at 20 Hz using QCapture Software (QImaging). Regions of interest (ROI) were selected where brightness changed significantly with contractions. Calcium changes were detected in the three ROIs indicated (Fig. 4A; in, on and outside the blocking electrode) and expressed as the fluorescent intensity ratio Δ*F*/*F* = (*F*−*F*_0_)/*F*_0_, in which *F* is the fluorescence and *F*_0_ is the minimum fluorescence detected during a typical AP. For time-lapse images and supplemental movie, drift on each pixel was first removed by subtraction of a linear fit over the entire recording, and each pixel was normalized to the maximum range of fluorescence measured during normal beating. Noise in each frame (300×400 pixels) was then reduced using a 6×6 median filter. Color range was purposefully set to span normal beating range, in order to highlight the wave propagation.

### Single-cell simulations

A mathematical model of the human atrial myocyte was used for the single-cell simulations [17]. The model was based on averaged voltage-clamp data recorded from isolated single myocytes and composed of a fluid compartment model including intracellular, cleft and extracellular spaces. It accounted for the changes in ionic concentrations in the cytoplasm as well as in the sarcoplasmic reticulum. The behavior of the cell was investigated under the case of transmembrane stimulation. The model was implemented in MATLAB® (The MathWorks), and high-frequency transmembrane current stimulation was simulated by adding the time-varying stimulation current to the total transmembrane current, following Nygren's implementation [17]. All simulations were performed by forward integration of the coupled system of differential equations. The temporal time step of the integration was chosen as 1 µs, and convergence of the results was validated using smaller time steps. Stimulations were simulated at frequencies between 5 Hz and 10 kHz with stimulus durations up to 5 s to match the experimental conditions. Typical simulation durations were 300–500 ms longer than stimulation duration to allow repolarization of the cell after the stimulus. The results obtained with AC inhibition stimulus were compared to the results of regular AP simulated with the model.

### Statistical analysis

Statistical data are shown as mean ± s.d. For analysis of significance, paired Student's *t*-test (repolarization analysis; Fig. 1E) was used and a *p*<0.05 was considered statistically significant. Significances were indicated with three stars for *p*<0.001 and two stars for *p*<0.01. The *n* values in the text and legends indicate the number of independent experiments (HL-1 cultures).

## Supporting Information

Figure S1 **Charge density per phase vs. frequency for square-wave, blocking stimuli (corresponding to** **Figure 1B** **in the main text).**
(TIF)

Figure S2 **Type-1 response – No response observed at low stimulation amplitudes.** Membrane potential is elevated slightly above resting level and oscillates synchronously with the stimulus. Stimulation parameters: *f* = 1 kHz, *I* = 2 nA. Stimulus applied between *t* = 20 ms and *t* = 5020 ms. Parameters are defined in the main text.
(TIF)

Figure S3 **Type-2 response – Single action potential.** Membrane potential is increased incrementally until an action potential is generated, after which the membrane oscillates synchronously with the stimulus around the slightly higher resting potential. Stimulation parameters: *f* = 1 kHz, *I* = 2.2 nA. Stimulus applied between *t* = 20 ms and *t* = 5020 ms. Parameters are defined in the main text.
(TIF)

Figure S4 **Type-3 response – Multiple action potentials.** Action potentials are generated sequentially when membrane potential is increased incrementally, until the threshold is reached. The membrane potential oscillates synchronously with the stimulus. Stimulation parameters: *f* = 1 kHz, *I* = 2.7 nA. Stimulus applied between *t* = 20 ms and *t* = 5020 ms. Parameters are defined in the main text.
(TIF)

Figure S5 **Type-4 response – Single prolonged action potential.** At high enough amplitudes, a single action potential with a prolonged plateau phase, sustained for the duration of stimulus, is generated. The membrane potential oscillates synchronously with the stimulus. Stimulation parameters: *f* = 1 kHz, *I* = 4.5 nA. Stimulus applied between *t* = 20 ms and *t* = 5020 ms. Parameters are defined in the main text.
(TIF)

Table S1 **Current thresholds for different type of responses obtained in single-cell simulations using square-wave stimulation.** For frequencies lower than 25 Hz, no prolonged action potential was observed, but the multiple action potential response persisted. All thresholds increase monolithically with increasing frequency. Simulations did not reveal the binary response observed in experiments. Thresholds are reported for a stimulus duration of 5 s.
(PDF)

Table S2 **Current thresholds for different type of responses obtained in single-cell simulations using sinusoidal wave stimulation.** For frequencies lower than 25 Hz, no prolonged action potential was observed, but the multiple action potential response persisted. All thresholds increase monolithically with increasing frequency. Thresholds for sinusoidal stimulation were higher compared to square-wave stimulation similar to experimental findings. Simulations did not reveal the binary response observed in experiments. Thresholds are reported for a stimulus duration of 5 s.
(PDF)

Movie S1 **Demonstration of local cardiac conduction block using high-frequency stimulation.** Blocking stimulus is applied between *t* = 5.7 s and *t* = 16.6 s. After a single action potential at the onset of the stimulus, the region defined by the stimulation electrode is decoupled from the rest of the syncytium, providing local conduction block. Red indicates high Ca^2+^ concentration, blue low.
(AVI)
