# Supplementary figures and images for: Spatiotemporally Controlled Cardiac Conduction Block Using High-Frequency Electrical Stimulation
Source: PLoS One. 2012 Apr 30;7(4):e36217. doi: 10.1371/journal.pone.0036217 (PMC3340354; doi:10.1371/journal.pone.0036217)

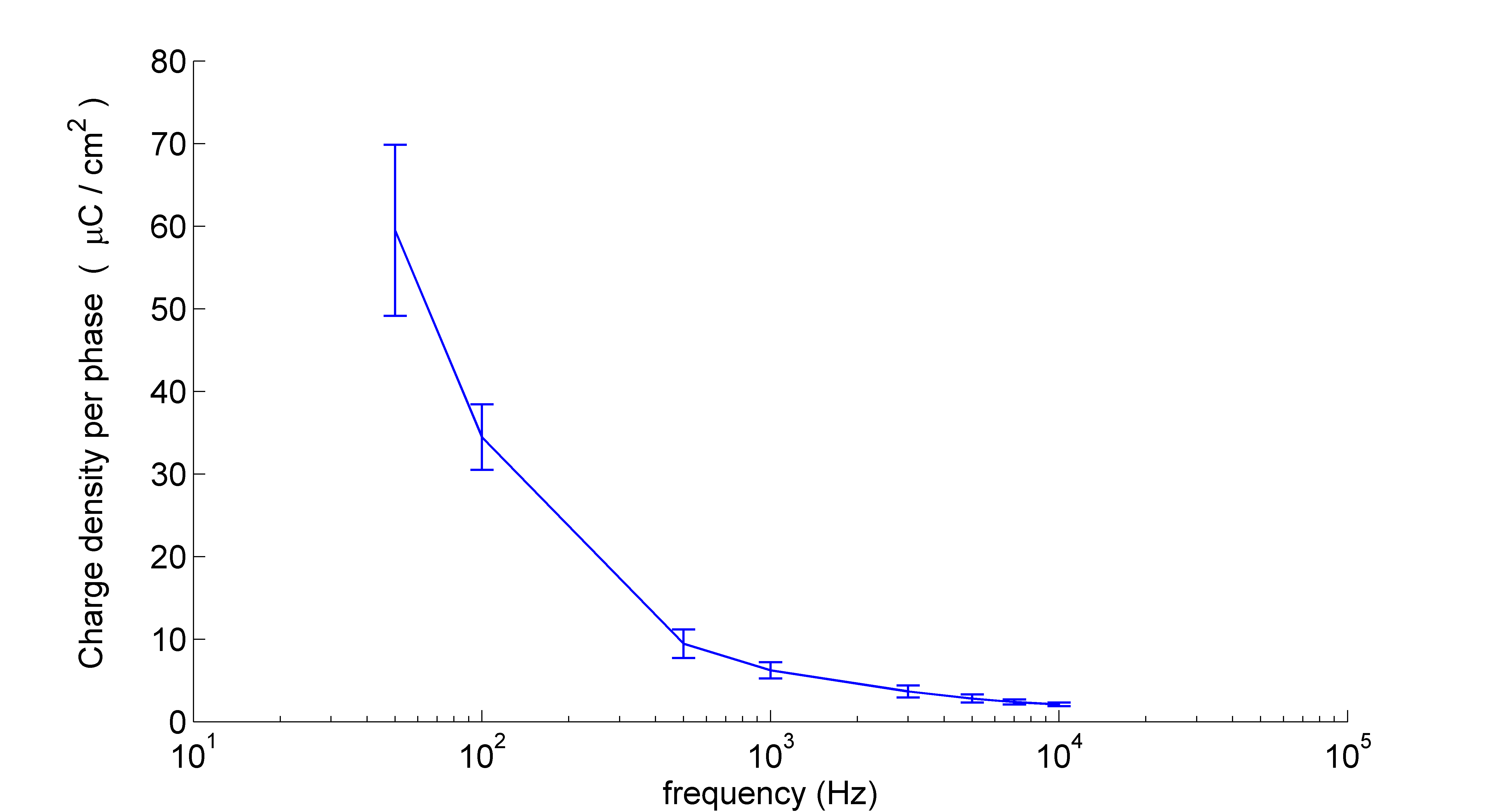

Supplement: Figure S1 — Charge density per phase vs. frequency for square-wave, blocking stimuli (corresponding to Figure 1B in the main text). (TIF) [file pone.0036217.s001.tif]

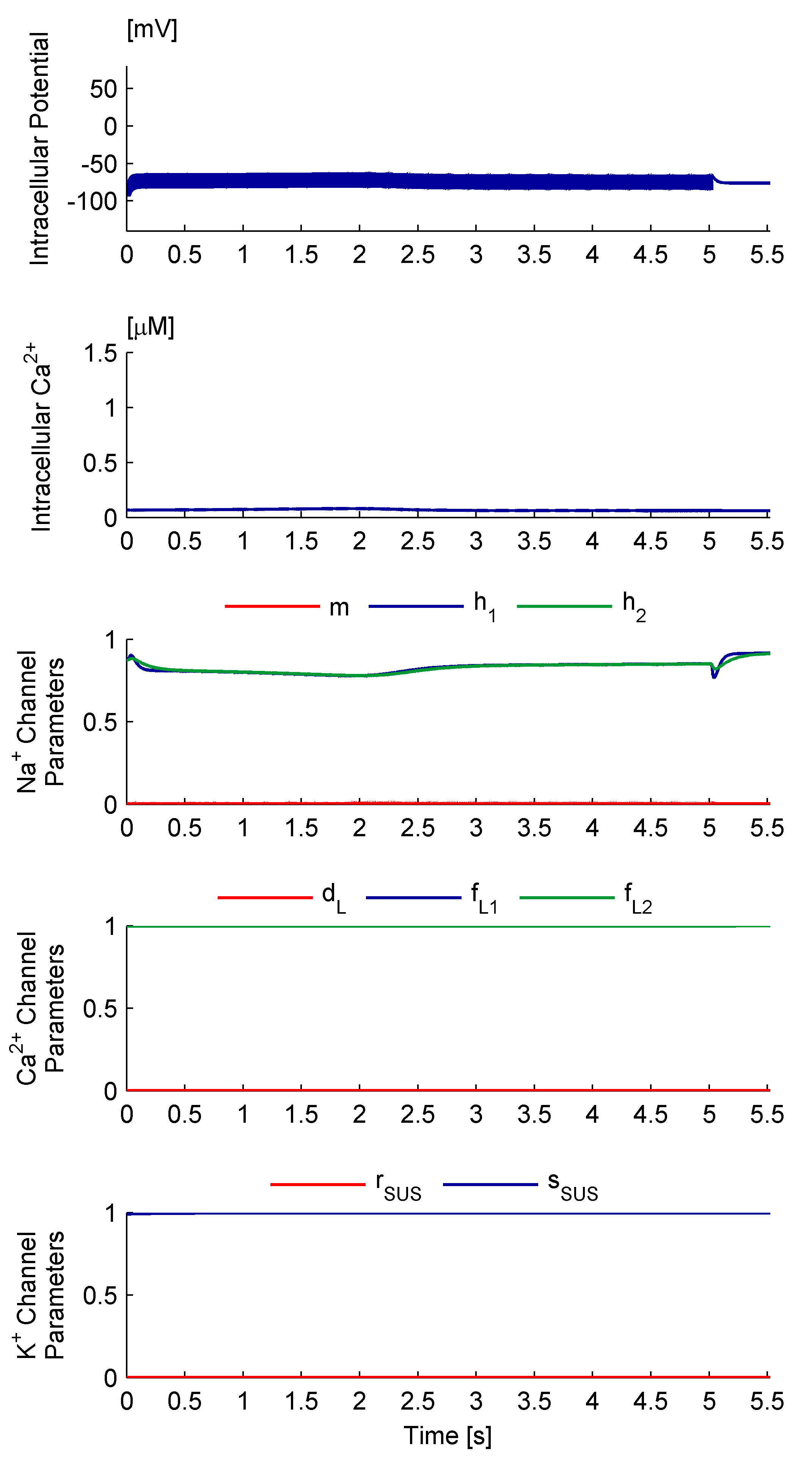

Supplement: Figure S2 — Type-1 response – No response observed at low stimulation amplitudes. Membrane potential is elevated slightly above resting level and oscillates synchronously with the stimulus. Stimulation parameters: f = 1 kHz, I = 2 nA. Stimulus applied between t = 20 ms and t = 5020 ms. Parameters are defined in the main text. (TIF) [file pone.0036217.s002.tif]

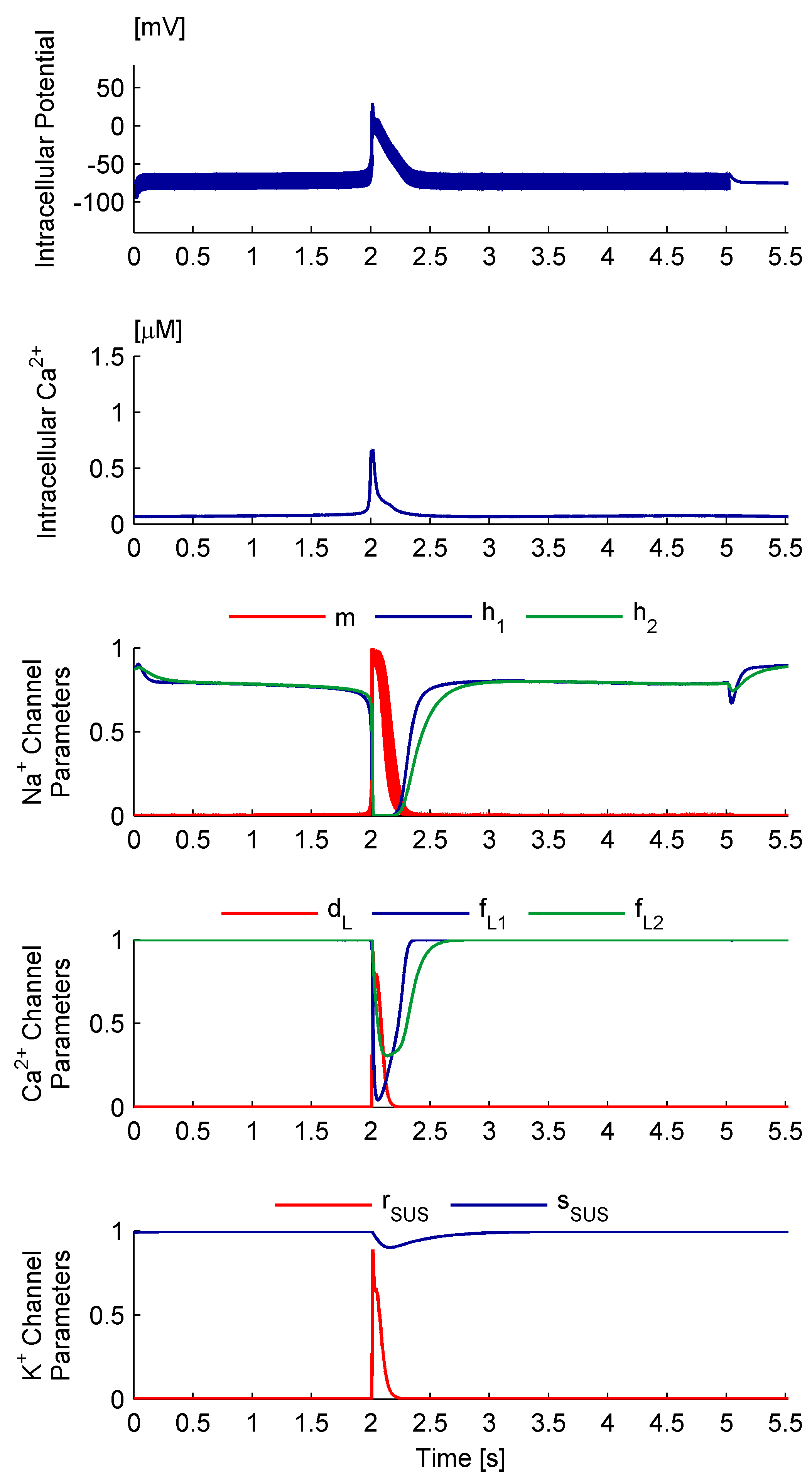

Supplement: Figure S3 — Type-2 response – Single action potential. Membrane potential is increased incrementally until an action potential is generated, after which the membrane oscillates synchronously with the stimulus around the slightly higher resting potential. Stimulation parameters: f = 1 kHz, I = 2.2 nA. Stimulus applied between t = 20 ms and t = 5020 ms. Parameters are defined in the main text. (TIF) [file pone.0036217.s003.tif]

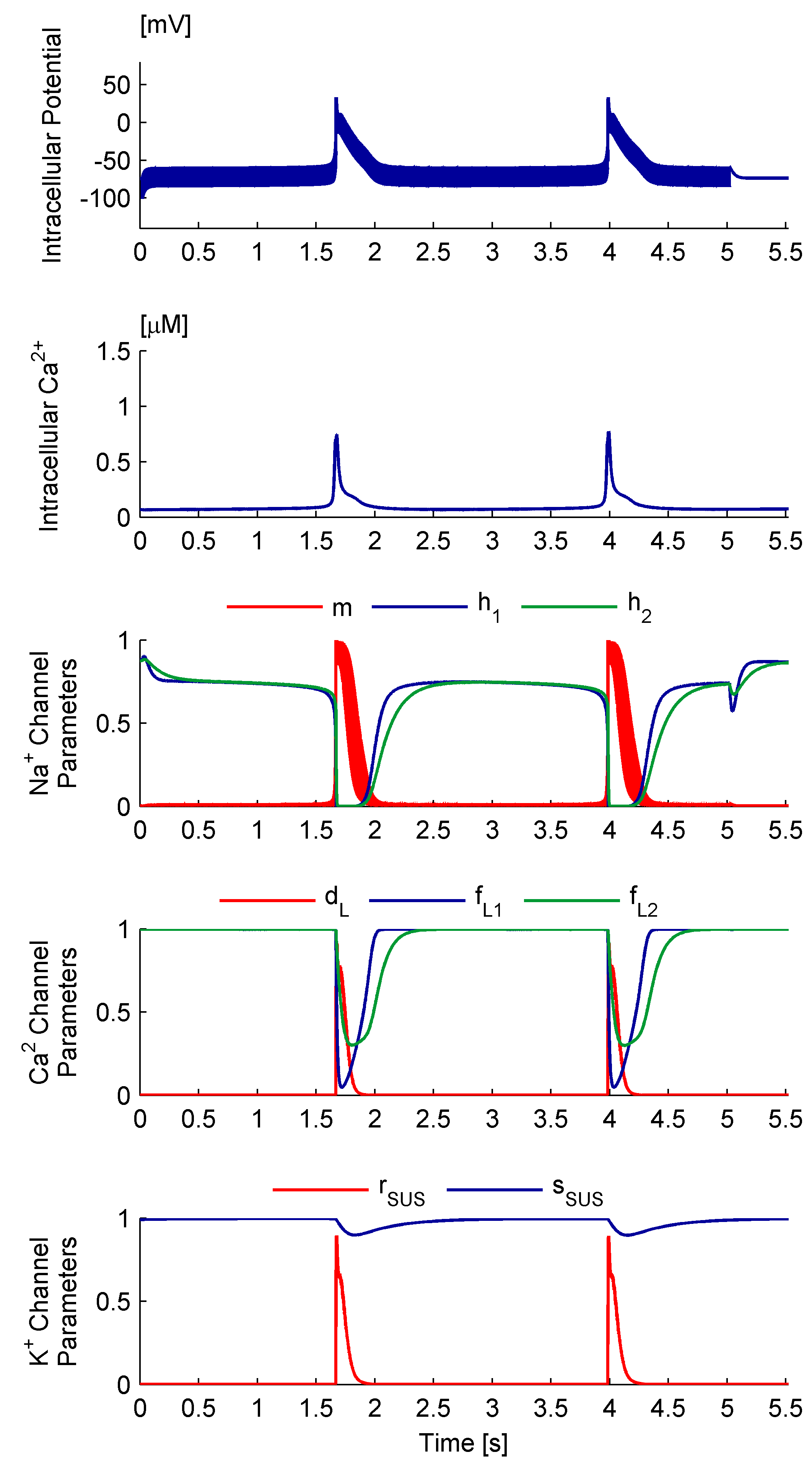

Supplement: Figure S4 — Type-3 response – Multiple action potentials. Action potentials are generated sequentially when membrane potential is increased incrementally, until the threshold is reached. The membrane potential oscillates synchronously with the stimulus. Stimulation parameters: f = 1 kHz, I = 2.7 nA. Stimulus applied between t = 20 ms and t = 5020 ms. Parameters are defined in the main text. (TIF) [file pone.0036217.s004.tif]

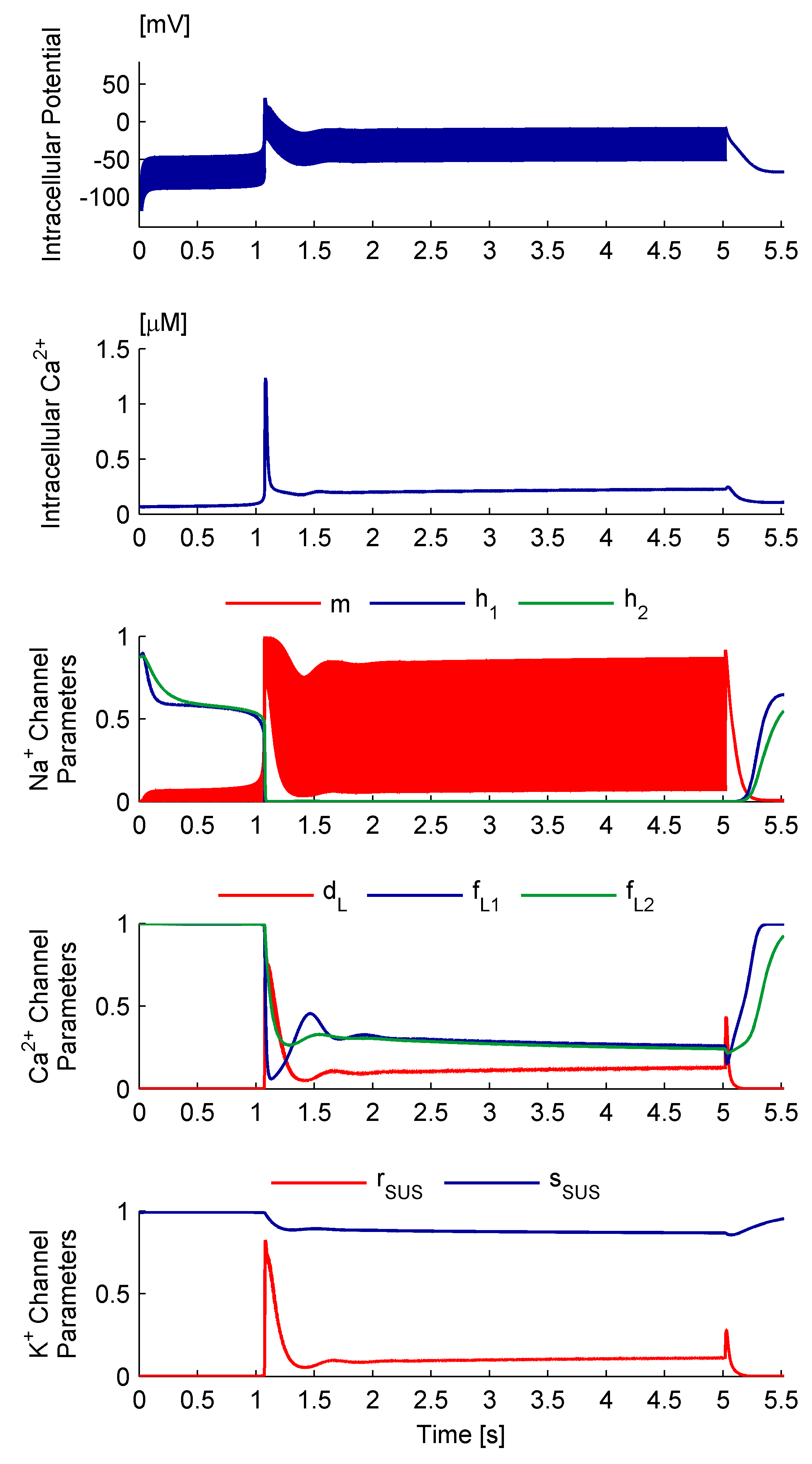

Supplement: Figure S5 — Type-4 response – Single prolonged action potential. At high enough amplitudes, a single action potential with a prolonged plateau phase, sustained for the duration of stimulus, is generated. The membrane potential oscillates synchronously with the stimulus. Stimulation parameters: f = 1 kHz, I = 4.5 nA. Stimulus applied between t = 20 ms and t = 5020 ms. Parameters are defined in the main text. (TIF) [file pone.0036217.s005.tif]
