# Supplementary material for: Spatiotemporally Controlled Cardiac Conduction Block Using High-Frequency Electrical Stimulation
Source: PLoS One. 2012 Apr 30;7(4):e36217. doi: 10.1371/journal.pone.0036217 (PMC3340354; doi:10.1371/journal.pone.0036217)
Supplement: Table S1 — Current thresholds for different type of responses obtained in single-cell simulations using square-wave stimulation. For frequencies lower than 25 Hz, no prolonged action potential was observed, but the multiple action potential response persisted. All thresholds increase monolithically with increasing frequency. Simulations did not reveal the binary response observed in experiments. Thresholds are reported for a stimulus duration of 5 s. (PDF) [file pone.0036217.s006.pdf]

	Current Thresholds (pA)		
Frequency (Hz)	Single AP	Multiple APs	Prolonged AP
5	16	21	-
10	25	30	-
25	55	70	90
50	110	130	160
100	210	250	320
250	520	600	800
500	1100	1300	1600
1000	2100	2500	3400
2000	4200	5200	7200
3000	6300	7600	12000
5000	11000	14000	23000
10000	21000	24000	47000

Table S1: Current thresholds for different type of responses obtained in single-cell simulations using square-wave stimulation. For frequencies lower than 25 Hz, no prolonged AP was observed, but the multiple-AP response persisted. All thresholds increase monolithically with increasing frequency. Simulations did not reveal the binary response observed in experiments. Thresholds are reported for a stimulus duration of 5 s.
