# Supplementary material for: Spatiotemporally Controlled Cardiac Conduction Block Using High-Frequency Electrical Stimulation
Source: PLoS One. 2012 Apr 30;7(4):e36217. doi: 10.1371/journal.pone.0036217 (PMC3340354; doi:10.1371/journal.pone.0036217)
Supplement: Table S2 — Current thresholds for different type of responses obtained in single-cell simulations using sinusoidal wave stimulation. For frequencies lower than 25 Hz, no prolonged action potential was observed, but the multiple action potential response persisted. All thresholds increase monolithically with increasing frequency. Thresholds for sinusoidal stimulation were higher compared to square-wave stimulation similar to experimental findings. Simulations did not reveal the binary response observed in experiments. Thresholds are reported for a stimulus duration of 5 s. (PDF) [file pone.0036217.s007.pdf]

| | Current Thresholds (pA) | | |
|---|---|---|---|
| Frequency (Hz) | Single AP | Multiple APs | Prolonged AP |
| 5 | 20 | 27 | - |
| 10 | 30 | 42 | - |
| 25 | 65 | 80 | 105 |
| 50 | 140 | 160 | 210 |
| 100 | 270 | 310 | 410 |
| 250 | 700 | 800 | 1100 |
| 500 | 1350 | 1550 | 2200 |
| 1000 | 2700 | 3100 | 4200 |
| 2000 | 5400 | 6200 | 9000 |
| 3000 | 8000 | 9300 | 16000 |
| 5000 | 14000 | 16000 | 27000 |
| 10000 | 27000 | 31000 | 60000 |

**Table S2:** Current thresholds for different type of responses obtained in single-cell simulations using sinusoidal wave stimulation. For frequencies lower than 25 Hz, no prolonged AP was observed, but the multiple-AP response persisted. All thresholds increase monolithically with increasing frequency. Thresholds for sinusoidal stimulation were higher compared to square-wave stimulation similar to experimental findings. Simulations did not reveal the binary response observed in experiments. Thresholds are reported for a stimulus duration of 5 s.
